# A study on the rapid diagnosis of chronic heart failure by measuring the difference in pulse oxygen saturation before and after arm compression

**DOI:** 10.1111/anec.13049

**Published:** 2023-02-27

**Authors:** Jian‐feng Jia, Li‐fei Wang, Xing‐li Gao

**Affiliations:** ^1^ Area in Department of Cardiology, Hypertension clinic The Ninth Hospital of Xingtai Xingtai China

**Keywords:** B‐type natriuretic peptide, chronic heart failure, diagnosis, pulse oxygen saturation

## Abstract

**Background:**

Clinically, the pulse oxygen saturation of patients with chronic heart failure does not decrease significantly, and the clinical manifestations of labor‐related dyspnea are not typical. As such, it is difficult to make a rapid diagnosis.

**Objective:**

To investigate changes in pulse oxygen saturation in patients with chronic heart failure and examine the relationship between B‐type natriuretic peptide (BNP) and normal pulse oxygen saturation.

**Methods:**

A total of 80 hospitalized patients with chronic heart failure and increased BNP were randomly selected as the study group; the family members of 60 patients without dyspnea were randomly selected as the control group. The researchers measured the value of pulse oxygen saturation before and after upper arm compression, calculating the difference and analyzing the correlation between this difference and BNP values. The data were statistically analyzed using the SPSS Statistics 17.0 program.

**Results:**

The decrease in pulse oxygen saturation in the study group was greater than in the control group; the decrease in pulse oxygen saturation of patients with chronic heart failure positively correlated with BNP.

**Conclusion:**

The value of pulse oxygen saturation in patients with chronic heart failure decreased more than in the control group, and this difference positively correlated with BNP. The measurement of pulse oxygen saturation before and after upper arm compression is a simple and effective method for diagnosing and evaluating chronic heart failure.

## Graphical abstract

Pulse oxygen saturation and B‐type natriuretic peptide (BNP) in patients with chronic heart failure. In this study, we found that the decreased value of pulse oxygen saturation in patients with chronic heart failure was greater than that of the normal control and correlated positively with the BNP level.

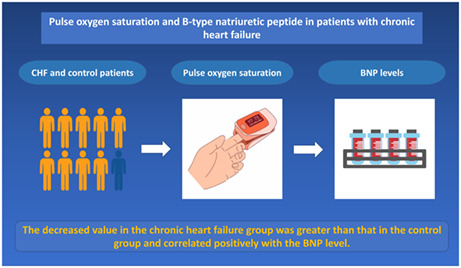

## 1 INTRODUCTION

B‐type natriuretic peptide (BNP) is a useful clinical biomarker for the diagnosis of heart failure (Suzuki & Sugiyama, 2018). Heart failure due to pulmonary congestion, or edema, can lead to pulmonary blood gas exchange disorders, often manifesting in the form of hypoxia and decreased arterial and pulse oxygen saturation. With a combination of labor dyspnea, coronary heart disease, and other basic symptoms, the level of BNP typically increases, making a clinical diagnosis more straightforward (Platz et al., 2017). However, there is no significant decrease in pulse oxygen saturation levels for patients with chronic heart failure (Hassan et al., 2021), and the clinical manifestations of labor‐related dyspnea are atypical. It is thus difficult to make a rapid diagnosis in clinical practice, and a BNP examination is often required to confirm the diagnosis. In addition, pulse oximetry is widely used in clinical practice because it can achieve noninvasive, continuous, and real‐time measurement of functional oxygen saturation (Levy et al., 2021; Pilcher et al., 2020). However, only a few studies have directly used pulse oxygen saturation to evaluate the severity of heart failure, particularly in patients with chronic heart failure (Sepehrvand et al., 2019). A recent study found that patients with heart failure of different severity had varying pulse oxygen saturation levels when measured using their fingers (Hassan et al., 2021). Accordingly, pulse oxygen saturation may not reflect the existence and extent of chronic heart failure.

To evaluate the severity of heart failure more accurately and quickly, this study used the cuff of a sphygmomanometer to pressurize the upper arm for 1 min. Pulse oxygen saturation was measured before and after the pressure test, which artificially caused ischemia and hypoxia of the upper limb. This value was anticipated to reflect the real oxygen saturation ability of the body. Afterward, the difference value was calculated using both results, which could estimate the potential degree of actual ischemia and hypoxia. This study also analyzed the relationship between this difference value and BNP and confirmed a significantly positive correlation, which may help to determine the degree and prognosis of heart failure according to pulse oxygen difference.

## 2 INFORMATION AND METHODOLOGY

### 2.1 General information

Between January and April 2020, in the first district of cardiology, 80 hospitalized patients with chronic heart failure with a quantitative increase in BNP were randomly selected as the study group. This group comprised 34 males and 46 females aged 51–95 years. In addition, 60 family members of patients without dyspnea were randomly selected during the same period as the control group. This group comprised 28 males and 32 females aged 45–89 years. The inclusion criteria for the study group were as follows: patients with chronic heart failure with BNP >100 pg/ml and clinical labor dyspnea. The exclusion criteria of the study group were as follows: acute left heart failure, moderate to severe pulmonary hypertension, shock, chronic obstructive pulmonary disease or pulmonary embolism, large area pulmonary infection, and sleep apnea hypopnea syndrome.

The inclusion criterion for the control group was as follows: no family members of patients with a dyspnea history. The exclusion criteria for the control group were as follows: a history of dyspnea, chronic bronchitis, bronchial asthma, lung disease, and sleep apnea hypopnea syndrome.

### 2.2 Method

This article represents a case–control observational study. Pulse oxygen saturation and BNP tests were performed on the same day. The following steps were taken: (1) The researchers measured the pulse oxygen saturation of the study and control group participants, respectively. A Yuyue brand finger pulse oxygen detector was used; the infrared ray was aimed at the skin above the nail, and the pulse oxygen of the right middle finger of the participants in the two groups was measured. Subsequently, an adult sphygmomanometer cuff was applied to the patient's arm and inflated for 1 min, pressurizing the side of the upper arm. The lower edge of the cuff was 3–4 cm above the cubital fossa, and the pressure was between 220 and 240 mmHg. After 1 min, the rapid ventilation was completed and the finger pulse oxygen level was measured immediately. The difference before and after was then calculated. (2) For the study group, BNP quantification was performed using the US‐manufactured med‐trec bedside rapid detection system, noting where BNP levels increased above 100 pg/ml. For each measurement, a BNP ratio was calculated by dividing the BNP level by the upper limit of normal for the assay used.

### 2.3 Evaluation indicators

The following evaluation indicators were used: (a) pulse oxygen saturation difference; (b) the correlation between pulse oxygen saturation difference and BNP.

### 2.4 Statistical analysis

The SPSS Statistics software program (v.17.0) was used for the statistical analysis. The normal distribution of the quantitative data was checked using a Kolmogorov–Smirnov test. Parametric tests were applied to data of normal distribution and nonparametric tests were applied to data of questionable normal distribution. Nominal data were compared using chi‐square tests; *p* < .05 was considered to indicate a statistical difference.

## 3 RESULTS

### 3.1 Baseline data

The average age of the control group was 66.93 ± 12.94, and the male‐to‐female ratio was 28/32. The average age of the study group was 71.23 ± 11.99, and the male‐to‐female ratio was 34/46. There were no significant differences regarding age and sex (*p* > .05). These results are shown in Table 1.

**TABLE 1:** Comparison of the difference of pulse/blood oxygen saturation

Group	Pulse oxygen saturation (before, %)	Pulse oxygen saturation (after, %)	Pulse oxygen saturation difference (%)
Control group (*n* = 60)	97.13 ± 1.50	96.67 ± 1.65	0.47 ± 1.25
Study group (*n* = 80)	97.33 ± 2.07	90.35 ± 8.29	6.98 ± 7.80
*t*	−0.429		
t^/^		4.695	−5.186
*p*	0.669	<0.001	<0.001

Abbreviations: *p*: *p* value; t/, Corrected *t*‐test value; *t*: *t*‐test value.

### 3.2 Comparison of pulse oxygen saturation between the two groups before and after the pulse oxygen experiment

Before the experiment, there was no significant difference in pulse oxygen saturation between the control group (97.13% ± 1.50%) and the study group (97.33% ± 2.07%) (*p* > .05). The two groups were thus comparable. After the experiment, there was a significant difference in pulse oxygen saturation between the control group (96.67% ± 1.65%) and the study group (90.35% ± 8.29%) (*p* < .001). The results are shown in Table 1.

### 3.3 Comparison of oxygen saturation differences between the two groups before and after the pulse oxygen experiment

A comparison between the control group (0.47% ± 1.25%) and the study group (6.98% ± 7.80%) showed that the pulse oxygen saturation difference in the study group was significantly larger (*p* < .001). The results are shown in Table 1.

### 3.4 Analysis of the correlation between pulse oxygen saturation difference and BNP in the study group

**FIGURE 1:**
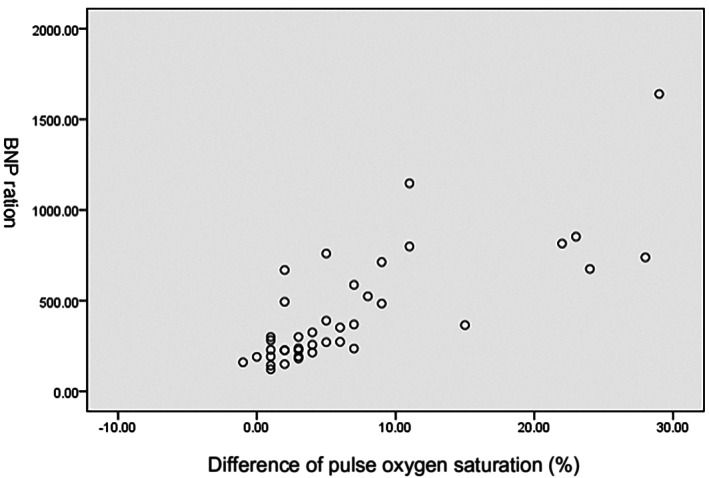
Scatter plot/correlation between BNP and pulse oxygen difference in study group

The Pearson correlation coefficient (*r* = .765, *p* < .05) showed that there was a positive correlation between the difference in pulse oxygen saturation and BNP quantification in the study group (see Figure 1).

## 4 DISCUSSION

Pulse oxygen saturation is measured using a fingertip oxygen probe, which is designed based on the oxygen absorption principle of red and infrared spectra. Studies have shown high consistency between arterial oxygen saturation and pulse oxygen saturation (Platz et al., 2017). Decreased pulse oxygen saturation reflects cardiovascular instability, heart failure, and increased risk of death (Figueroa & Peters., 2006). In a previous study, an acute coronary event registration score showed that pulse oxygen saturation decreased significantly in the acute coronary event group (Jun & Zheng., 2016). In another study, a negative correlation was found between 6 month mortality and pulse oxygen saturation during hospitalization in patients with heart failure (Yanping et al., 2009). Studies have also shown that the long‐term management of patients with chronic heart failure using remote pulse oxygen measurement can lead to the early detection of increased heart failure (Xiaolin et al., 2013). Therefore, it is easy to determine the oxygen saturation state of the body from pulse oxygen measurements, which is beneficial for assessing heart failure and prognosis (Li Qiuru & Minghua., 2013). However, the abovementioned studies have limited benefits for the management and follow‐up of chronic heart failure after discharge. The pulse oxygen saturation of hospitalized patients with chronic heart failure is not improved by drugs, oxygen inhalation, long‐term compensation, and other treatments. Currently, the limitations of oxygen saturation measurement are obvious. This study examined how to remove these effects and use pulse oxygen saturation to evaluate the state of heart function and the degree of heart failure during hospitalization. The arterial blood supply of the upper limb was temporarily blocked by inflation and compression of the upper limb cuff, which artificially caused ischemia and hypoxia of the upper limb. When the arterial blood flowed again after rapid decompression, oxygen diffused to the surrounding tissue, and the fingertip pulse oxygen level was measured quickly. At this time, the value should have reflected the real oxygen saturation ability of the body, and the differences in the results before and after this experiment represented the potential degree of ischemia and hypoxia. This study was conducted according to the above reasoning and successfully confirmed this hypothesis. The difference in pulse oxygen saturation was obvious in patients with chronic heart failure. Additionally, B‐type natriuretic peptide is also an important index for diagnosing and assessing the degree of heart failure. In this study, patients with chronic heart failure and an increased BNP were selected as research participants. An analysis of the relationship between pulse oxygen saturation difference and BNP quantification was carried out, with the results showing a positive correlation. This indicated that pulse oxygen saturation difference could reflect the state of BNP, which could help to identify the condition and prognosis of patients (Meifang & Shuzhi, 2018).

Oxygen saturation is the percentage of the actual binding oxygen of hemoglobin and the maximum binding oxygen of hemoglobin. It is an important indicator of lung blood gas exchange, arterial oxygen partial pressure, tissue oxygen consumption, and cardiac output. The blood gas exchange disorder and decreased cardiac output caused by pulmonary congestion and edema in chronic heart failure inevitably lead to a decrease in oxygen partial pressure and oxygen saturation. Oxygen inhalation, drug treatment, and body compensation can make the surface of pulse oxygen saturation normal. However, when the upper arm is pressurized and the arterial blood flow is temporarily blocked to cause artificial upper arm ischemia and hypoxia, the essence of the decrease in arterial oxygen partial pressure and oxygen saturation is restored. This can indirectly and truly reflect the degree of blood gas exchange disorder in the lungs and is conducive to determining the degree of heart failure.

This study has some limitations. The ejection fraction was not included in the observation index, and the observation range was narrow. In addition, the sample size was small.

In conclusion, the detection of pulse oxygen saturation after upper arm compression has significant value in the diagnosis and evaluation of heart failure.

## AUTHOR CONTRIBUTIONS

JJF conceived of the study, and WLF and GXL participated in its design and coordination and helped to draft the article. All authors read and approved the final manuscript.

## FUNDING INFORMATION

Xingtai Science and Technology Support Plan Project of Xingtai Science and Technology Bureau: 2019ZC308.

## CONFLICT OF INTEREST STATEMENT

All the authors had no personal, financial, commercial, or academic conflicts of interest separately.

## ETHICS APPROVAL AND CONSENT TO PARTICIPATE

This study was conducted in accordance with the Declaration of Helsinki and approved by the ethics committee of The Ninth Hospital of Xingtai. All participants signed an informed consent form for inclusion in the study.

## CONSENT FOR PUBLICATION

Not applicable.

## Data Availability

All data generated or analyzed during this study are included in this published article.
